# A Preliminary Investigation of a Conceptual Model Describing the Associations Between Childhood Maltreatment and Alcohol Use Problems

**DOI:** 10.3390/brainsci14111081

**Published:** 2024-10-29

**Authors:** Nayani Ramakrishnan, Sujaiya Tiba, Abby L. Goldstein, Suzanne Erb

**Affiliations:** 1 Department of Psychology, University of Toronto Scarborough, 1265 Military Trail, Toronto, ON M1C 1A4, Canada; sujaiya.tiba@mail.utoronto.ca (S.T.); suzanne.erb@utoronto.ca (S.E.); 2 Department of Applied Psychology and Human Development, Ontario Institute for Studies in Education, University of Toronto, 252 Bloor Street West, Toronto, ON M5S 1V6, Canada; abbyl.goldstein@utoronto.ca

**Keywords:** childhood maltreatment, alcohol use problems, emotion dysregulation, reward processes, impulsivity

## Abstract

Background/Objectives: Childhood maltreatment has been linked to numerous adverse outcomes in adulthood, including problem substance use. However, not all individuals exposed to childhood maltreatment develop substance use problems, indicating the role of other factors in influencing this outcome. Past work suggests that adverse early life experiences, including childhood maltreatment, lead to neurobiological changes in frontolimbic functions that, in turn, result in altered stress and reward responses, heightened impulsivity, affect dysregulation, and, ultimately, increased risk for maladaptive behaviors such as substance use. The aim of this preliminary investigation using cross-sectional data was to test associations between these factors in the relationship between childhood maltreatment and alcohol use problems in a sample of emerging adults. Methods: Emerging adults (18–30 years old) who identified as regular drinkers (i.e., drinking at least 2–4 times in the past month) were recruited from a crowd-sourcing platform (Prolific) as well as community samples. Participants completed online standardized questionnaires assessing reward sensitivity and responsiveness, impulsivity, emotion regulation, and alcohol consequences. Results: Path analyses demonstrated good fit for the data (SRMR = 0.057, RMSEA = 0.096, 90% CI [0.055, 0.142], CFI = 0.957). Childhood maltreatment was associated with reward responsiveness (β = −0.026, Z = −4.222, *p* < 0.001) and emotion dysregulation (β = 0.669, Z = 9.633, *p* < 0.001), which in turn was associated with urgency and, subsequently, alcohol consequences (β = 0.758, Z = 7.870, *p* < 0.001). Conclusions: Although these findings are preliminary, the current study is one of the first to test a comprehensive model addressing the relationship between childhood maltreatment and alcohol use problems. The findings have the potential to inform treatment strategies that target motivation and goal-directed action for reducing and managing consequences associated with childhood maltreatment. Future research should test the model using longitudinal data to address the limitations of a cross-sectional study and assess temporal associations between constructs.

## 1. Introduction

Childhood maltreatment is broadly defined as exposure to physical, emotional, and/or sexual abuse and/or physical and emotional neglect during childhood or adolescence [1]. Moreover, childhood maltreatment that occurs during critical developmental windows increases the risk of unhealthy coping, risk taking, and psychopathology in adulthood [2,3,4], including substance use problems in emerging adulthood [5,6,7]. The occurrence of childhood maltreatment is prevalent in society, with more than 60% of adults in the U.S. (BRFSS, 2011–2020; [8]) and Canada [9] reporting some form of early life adversity. 

Childhood threat and childhood deprivation have been identified as representing two distinct underlying dimensions of childhood maltreatment that, while not exhaustive in terms of capturing all types of maltreatment, can distinguish different forms of adversity in instructive ways [10,11,12]. Childhood threat refers to experiences that represent a threat to one’s physical integrity (i.e., instances of harm or the potential for harm), and childhood deprivation refers to the absence of expected environmental inputs (i.e., lack of cognitive, social, and physical inputs commonly found in caregiver interactions). Evidence from neurobiological studies has revealed that childhood threat and deprivation have different effects on the brain and may lead to different functional outcomes, including cognitive and emotional outcomes [12,13]. For example, a longitudinal study conducted by Miller and colleagues (2018) [11] revealed an indirect effect of verbal abilities on the relationship between childhood deprivation and externalizing but not internalizing problems, suggesting that unique developmental processes may link specific types of adversity and problem outcomes. Relatedly, other studies have found that childhood deprivation, but not childhood threat, is associated with lower executive function in early childhood [14,15] and that childhood threat (i.e., betrayal) is strongly associated with an increased probability of subsequent substance use and other disorders [16]. Although differential impacts of threat vs. deprivation have been identified in the literature, there is limited research on how these distinct forms of childhood maltreatment influence the manner in which substance use problems develop.

While childhood maltreatment has been strongly linked to substance use problems, this outcome does not occur in all cases, suggesting that other factors influence the relationship [17]. Indeed, impairment in reward processes (e.g., blunted reward responsiveness during the anticipation of rewards [18]), heightened trait impulsivity and poor impulse control [19,20], and emotion dysregulation [21,22] have each been identified as factors that increase risk for the development of substance use problems in the context of childhood maltreatment. To date, however, there is little research on how these underlying mechanisms interact to influence the pathways from childhood maltreatment to substance use problems.

One of the first conceptual models describing a pathway from early life adversity to the development of maladaptive behaviors such as substance use problems was proposed by Lovallo in 2013. By Lovallo’s account [23], childhood maltreatment contributes to neurobiological changes in adrenocortical and endogenous opioid systems, in turn leading to modified frontolimbic functions, including reduced stress responses, heightened impulsive responses, and unstable affect regulation (i.e., increased negative affect). Ultimately, according to Lovallo, these changes lead to an increased propensity for maladaptive behaviors such as problem substance use and poor health outcomes.

Extending on Lovallo’s (2013) model, al’Absi (2020) [23,24] proposed a modified conceptual model that addresses the temporal relationship between factors influencing the relationship between childhood maltreatment and problem substance use [22]. More specifically, al’Absi (2020) [24] suggested that exposure to childhood maltreatment heightens emotional reactivity to environmental cues. In parallel, it leads to a diminished response to rewarding cues and an increased propensity for seeking rewards. These changes, in turn, impact decision making, leading to poor impulse control, heightened engagement in impulsive behaviors, and, ultimately, increased problem substance use.

Thus, based on the models that have been proposed to date, there are three major factors that influence the relationship between childhood maltreatment and substance use problems: emotion regulation, reward-related processes, and impulsivity. Emotion regulation can be broadly defined as the ability to manage one’s own emotional experiences and expressions, often in the context of negative emotions. Individuals with experiences of childhood maltreatment often have difficulties with emotion regulation, which has been linked to increased psychopathology [20]. Moreover, individuals with high levels of affect reactivity are susceptible to more intense negative affect and greater use of maladaptive strategies to cope with this negative affect [23]. In regard to reward-related processes, problems associated with substance use are exacerbated when information related to the processing of rewards is disrupted as a result of childhood maltreatment. For example, childhood maltreatment has been found to increase reward responsivity, and the hyper-responsivity of the reward circuitry increases the risk for future substance use [25]. And finally, impulsivity, a multidimensional construct associated with the tendency to act rashly without thought of future consequences (i.e., response disinhibition [26]), has been found to indirectly influence the relationship between childhood maltreatment and problems related to alcohol and cannabis use in young adults [27,28,29,30,31].

Building on past work from our lab [32] and in alignment with al’Absi’s (2020) [24] heuristic model of the relationship between childhood maltreatment and substance use problems, the current cross-sectional investigation was carried out to empirically examine associations between emotion dysregulation, reward processing, and impulsivity in mediating the effects of childhood maltreatment on problem substance use, more specifically on alcohol-related problems. Using a cross-sectional, integrated model, this is one of the first studies to empirically test associations between risk factors (reward, emotional dysregulation, and impulsivity), childhood maltreatment, and alcohol use problems. While these particular factors have been examined separately in previous research, we are unaware of any other research using an integrated analysis to capture combined associations. Moreover, by investigating how this model may vary based on participant characteristics, this study has the potential to inform individualized intervention and prevention strategies aimed at mitigating the effects of childhood maltreatment on alcohol use problems. 

Study participants represented a sample of “emerging” adults (18–30 years old), and the outcome variables focused specifically on alcohol use problems. Based on data from North America, alcohol is the most commonly used substance among emerging adults [33,34], and relative to other age groups, emerging adults experience the highest rates of alcohol and other substance use problems [35,36,37]. Moreover, emerging adults with a history of childhood maltreatment demonstrate earlier initiation and excessive consumption of alcohol, including more episodes of binge drinking and an increased risk for alcohol use disorder, compared to those who do not report experiencing early life stress [38,39,40,41].

## 2. Materials and Methods

### 2.1. Participants and Procedures

Emerging adults (*n* = 397) aged 18–30 (M = 24.92; SD = 2.99) were recruited from the community using Prolific (a crowdsourcing platform; *n* = 351) or Kijiji (*n* = 46). Informed consent was obtained, and participants were asked to complete a screening questionnaire on Qualtrics. To ensure that the sample included current drinkers, participants were considered eligible if they reported drinking at least 2–4 times in the past month.

Participants completed a set of self-report questionnaires through Qualtrics over an approximately 30–45 min period. Written instructions pertaining to the questionnaires were administered online. Research assistants were available to answer questions and/or concerns. All study procedures were approved by the University of Toronto Research Ethics Boards (REB #40003).

### 2.2. Measures

#### 2.2.1. Demographics Questionnaire

Participants began by completing a demographics questionnaire assessing age, biological sex, gender identity, ethnicity, religion, education (completed to date), occupation (if applicable), socioeconomic status as determined by household income and level education of completed by parents, relationship status, and any current or past diagnosis of a mental health disorder (including treatments or medications).

#### 2.2.2. Childhood Maltreatment

The Childhood Trauma Questionnaire—Short Form (CTQ; [1,42]) is a 28-item retrospective self-report questionnaire that assesses exposure to five categories of childhood maltreatment experiences: physical abuse, emotional neglect, emotional abuse, physical neglect, and sexual abuse. Each subscale consists of five items, and participants indicated the degree to which each item (e.g., “I got hit so hard that I had to see a doctor or go to the hospital”) was true for them “while growing up” on a 5-point Likert scale, ranging from 1 (never) to 5 (very often). High reliability and internal consistency reliability have been demonstrated for the CTQ across various samples [1]. The total CTQ score (i.e., the sum of scores across all the subscales) was calculated for the current study and is often used in the literature to reflect severity of childhood maltreatment [43]. Subscales assessing childhood threat (physical, emotional, and sexual abuse) and deprivation (physical and emotional neglect) were also computed (i.e., the sum of scores across the subscales). Cronbach’s alpha for the total score was 0.88, for childhood threat was 0.90, and for childhood deprivation was 0.92.

#### 2.2.3. Reward Processes

The Temporal Experiences Scale (TEPS; [44]) is an 18-item self-report questionnaire that assesses trait anticipatory pleasure (10 items) and consummatory pleasure (8 items). The measure utilizes a 6-point Likert scale, ranging from 1 = very false for me to 6 = very true for me. Reward anticipation is related to responsiveness and behavioral activation (e.g., “When I think about eating my favorite food, I can almost taste how good it is”), whereas reward consumption examines the satisfaction a person feels when they receive a reward (e.g., “I enjoy taking a deep breath of fresh air when I walk outside”). Consistent with other studies, Cronbach’s alpha for the current study for reward anticipation was 0.78 and for reward consumption was 0.72.

The Sensitivity to Punishment and Reward Questionnaire (SPSRQ; [45]) is a 48-item self-report questionnaire developed to assess two subconstructs: sensitivity to reward (e.g., “Does the good prospect of obtaining money motivate you strongly to do some things?”) and sensitivity to punishment (e.g., “Do you often refrain from doing something because you are afraid of it being illegal?”), with each subconstruct consisting of 24 items. Participants respond with a 0 = no or 1 = yes for each item, which is then summed to create a total score for each facet. The SPSRQ has been shown to have adequate internal consistent and test–retest reliability [45,46]. Cronbach’s alpha for the current study for sensitivity to reward was 0.79 and sensitivity to punishment was 0.87.

Behavioral Activation System and Behavioral Inhibition System Scales (BAS/BIS; [47]) are used to assess two systems that govern approach and avoidance behavior, respectively. More specifically, the BAS is associated with reward-seeking behavior and approach motivation, while the BIS is associated with inhibition, anxiety, and avoidance. The BAS consists of 13 items with three subconstructs—reward responsiveness (e.g., “When I am doing well at something I love to keep at it”), fun seeking (e.g., “I am always willing to try something new if I think it will be fun”), and drive (e.g., “I go out of my way to get things I want”). The BIS consists of seven items (e.g., “Even if something bad is about to happen to me, I rarely experience fear or nervousness”) and is computed as a total score. Cronbach’s alpha for the current study for BAS reward responsiveness was 0.69, for BAS fun seeking was 0.74, for BAS drive was 0.80, and for the BIS was 0.84.

#### 2.2.4. Impulsivity

The Short UPPS-P Impulsive Behavioral Scale (SUPPS-P; [27]) is a 20-item self-report questionnaire that measures personality facets associated with impulsivity. The measure, which was developed using the original 59-item UPPS-P questionnaire by converging common traits of impulsivity [48,49,50], is comprised of five subscales: negative urgency (the tendency to experience strong impulses in response to negative mood), positive urgency (the tendency to experience strong impulses in response to positive mood), (lack of) premeditation (the tendency to act without considering the consequences), (lack of) perseverance (the inability to stay focused), and sensation seeking (the tendency to pursue exciting activities and an openness to new experiences). Participants indicate the degree to which each statement (e.g., “I tend to act without thinking when I am really excited.”) is true on a 4-point Likert scale ranging from 1 (low level of self-reported impulsivity) to 4 (high level of self-reported impulsivity). The SUPPS-P subscales have good internal consistency, with alpha ranging from 0.74 to 0.88 across the five subscales, and it is strongly correlated with the subscales of the UPPS-P [27]. In the current sample, Cronbach’s alpha for the subscales are as follows: negative urgency—0.79, positive urgency—0.83, sensation seeking—0.68, (lack of) premeditation—0.79, and (lack of) perseverance—0.68.

#### 2.2.5. Emotion Regulation

The Difficulties in Emotion Regulation Scale (DERS; [51]) is a 36-item self-report widely used measure that assesses an individual’s levels of emotion dysregulation across six domains: nonacceptance of emotional responses, difficulty engaging in goal-directed behavior, impulse control difficulties, lack of emotional awareness, limited access to emotion regulation strategies, lack of emotional clarity, and strategies for emotion regulation. Examples of items include “I pay attention to how I feel” and “When I’m upset, I feel out of control”. Participants indicate their responses on a 5-point Likert scale ranging from “almost never” to “almost always”. For the purposes of the current study, a total score across all six domains was computed. The DERS has consistently shown strong test–retest reliability [52]. In the current sample, Cronbach’s alpha was 0.95.

#### 2.2.6. Alcohol Use Consequences

The Brief Young Adult Alcohol Consequences Questionnaire (B-YAACQ; [53,54]) is a self-report questionnaire that examines the harmful consequences related to alcohol consumption in young adults. The B-YAACQ consists of 24 yes/no items. Examples of items include “While drinking, I have done or said embarrassing things” and “I have had a hangover (headache, sick stomach) the morning after I had been drinking.”. The B-YAACQ has demonstrated strong reliability and validity. In the current sample, Cronbach’s alpha was 0.91.

### 2.3. Data Analytic Plan

#### 2.3.1. Testing for Indirect Effects

Due to the multi-dimensional nature of the constructs included in the study (emotion dysregulation, reward processing, and impulsivity) and the past research indicating that these constructs may interact differentially with childhood maltreatment factors (e.g., [55,56,57,58,59]) and substance use problems (e.g., [60,61,62]), it was important to determine which dimensions (i.e., facets) and corresponding subscales best represented each of the measured constructs. Preliminary analyses included conducting five separate tests of indirect effects using PROCESS macro v3.5 for SPSS 26 (model 4; [63]) to test which facet(s) from each of the multidimensional constructs indirectly influences the relationship between childhood maltreatment and alcohol use problems. For reward processes, models included an estimate of the indirect effect of childhood maltreatment on alcohol use problems through facet scores of the TEPS (i.e., reward anticipation and reward consumption), SPSRQ (i.e., sensitivity to punishment and sensitivity to reward), BAS (i.e., reward responsiveness, fun seeking, drive) and the BIS total score. For impulsivity, models included an estimate of the indirect effect of childhood maltreatment on alcohol use problems through facet scores of the SUPPS-P (i.e., positive and negative urgency, sensation seeking, lack of premeditation, and lack of perseverance). Bias-corrected 95% bootstrap confidence intervals (CI) were estimated for all indirect effects. Indirect effects were deemed significant if the 95% bootstrap CI did not contain zero.

#### 2.3.2. Testing the Full Conceptual Model

Once significant facets were identified, tests of the conceptual model were conducted using path analysis in R version 4.2.2. with the lavaan package 0.6–12 [64]. Childhood maltreatment (i.e., total CTQ score) was modeled as an exogenous variable. The endogenous variables were the significant facets (as identified by the initial tests for indirect effects) for reward processes and impulsivity along with emotion dysregulation (total DERS score) and, finally, alcohol use problems (i.e., total YAACQ scores). Finally, to test whether the model changed when threat (physical, emotional, and sexual abuse) and deprivation (physical and emotional neglect) were considered separately, individual models for both dimensions were also conducted.

Based on the recommendations of Pavlov and colleagues (2021), goodness-of-fit indices were used to determine an approximate representation of the conceptual model [65]. The standardized root mean squared residual (SRMR), which assesses the magnitude of the observed and expected correlations (acceptable range of between 0.05 and 0.08; [66]), was chosen as the absolute goodness-of-fit measure. The root mean squared error of approximation (RMSEA), which assesses the extent to which the hypothesized model deviates from a perfect model (values between 0.08 and 0.1 considered marginal; [67]), was chosen to represent model misfit (i.e., “badness of fit”). Finally, the comparative fit index (CFI) was chosen as a measure of relative goodness of fit (acceptable model fit is CFI value of 0.95 or greater [66]).

## 3. Results

### 3.1. Participant Demographics and Descriptive Data

Most of the sample reported their biological sex as female (59.4%). Additional demographic characteristics of the participants, such as racial background and socioeconomic status (i.e., household income and education completed by parents), are presented in Table 1.

Descriptive data and bivariate correlations for childhood maltreatment (CTQ), emotion dysregulation (DERS), and alcohol use problems (B-YAACQ) as well as the subscales of the reward and impulsivity measures that selectively indirectly influenced the relationship between childhood maltreatment and alcohol use problems (see Indirect Effects below) are presented in Table 2.

### 3.2. Planned Tests of Indirect Effects

Tests of all direct and indirect effects of the constructs of interest are presented in Table 3. As predicted, there was a significant direct effect of childhood maltreatment on alcohol use problems (b = 0.120, SE = 0.024, 95% CI [0.074, 0.166]).

Three separate indirect effect models that varied according to the reward-related indices revealed that only the reward responsiveness subscale from the BAS indirectly influenced the relationship between childhood maltreatment and alcohol use problems (b = 0.010, SE = 0.0006, 95% CI [<0.001, 0.023]; Table 3). In addition, and consistent with our hypotheses, negative urgency (b = 0.027, SE = 0.009, 95% CI [0.012, 0.048]) and positive urgency (b = 0.017, SE = 0.008, 95% CI [0.004, 0.035]) from the SUPPS-P indirectly influenced the relationship between childhood maltreatment and alcohol use problems (Table 3).

### 3.3. Path Analyses

Based on the results of our planned tests of indirect effects, we tested the overall conceptual model by carrying out separate path analyses for the SUPPS-P subscales of negative and positive urgency, each of which represents a unique facet of impulsivity. We also ran separate path analyses to test associations with the total CTQ score and with the threat and deprivation subscores; in all path analyses, reward responsiveness from the BAS and the total scores from the DERS were entered for the construct measures of reward and emotion dysregulation, respectively.

**Figure 1:**
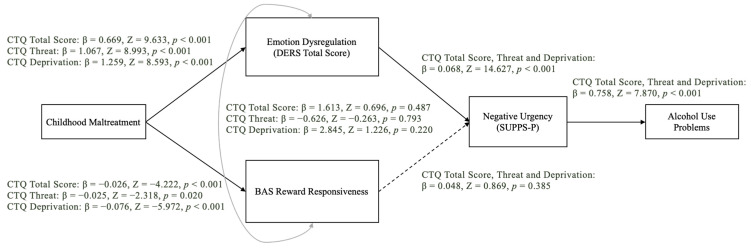
Path model depicting the association between childhood maltreatment and alcohol use problems through emotion dysregulation, reward responsiveness, and negative urgency. Model fits for CTQ total [SRMR = 0.057, RMSEA = 0.096, 90% CI [0.055, 0.142], CFI = 0.957]; CTQ threat [SRMR = 0.050, RMSEA = 0.096, 90% CI [0.055, 0.142], CFI = 0.954]; and CTQ deprivation [SRMR = 0.050, RMSEA = 0.081, 90% CI [0.039, 0.128], CFI = 0.969]. The dashed arrow represents a non-significant association.

**Figure 2:**
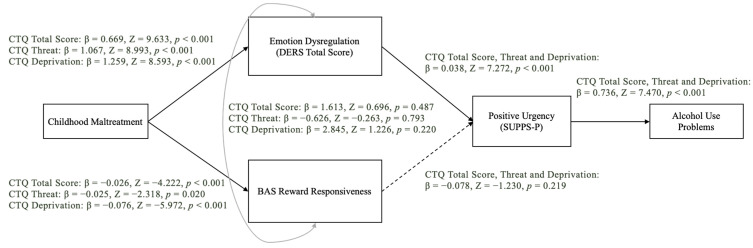
Path model depicting the association between childhood maltreatment and alcohol use problems through emotion dysregulation, reward responsiveness, and positive urgency. Model fits for CTQ total [SRMR = 0.079, RMSEA = 0.132, 90% CI [0.092, 0.177], CFI = 0.878]; CTQ threat [SRMR = 0.077, RMSEA = 0.130, 90% CI [0.089, 0.174], CFI = 0.869]; and CTQ deprivation [SRMR = 0.073, RMSEA = 0.125, 90% CI [0.085, 0.170], CFI = 0.890]. The dashed arrow represents a non-significant association.

In all models, negative urgency demonstrated a good fit for the conceptual model (see Figure 1 for respective model fit indices). Child maltreatment was related to reward responsiveness and emotion dysregulation; however, only emotion dysregulation was associated with negative urgency. Finally, negative urgency was associated with alcohol use problems (see Figure 1 for all direct effects). In contrast, the path analysis for positive urgency did not demonstrate good fit according to the model fit indices we adopted a priori (see Figure 2). That said, similar to the analysis for negative urgency, child maltreatment was associated with reward responsiveness and emotion dysregulation, while emotion dysregulation only was associated with positive urgency. Finally, and as was the case for negative urgency, positive urgency was associated with alcohol use problems (see Figure 2 for all direct effects).

It is important to note that although the RMSEA values for the second model suggest a marginal fit, they cannot be interpreted in isolation. Other goodness-of-fit indices should also inform decisions about model fit, including the SRMR and CFI, which were within the good-to-acceptable range. Furthermore, according to Chen et al., 2008, using a cutoff value of 0.05 for the RMSEA may result in rejecting valid models when sample sizes are relatively small [68].

## 4. Discussion

To our knowledge, this study is the first to empirically test in a single model the indirect effects of risk factors of reward, emotion dysregulation, and impulsivity on the association between childhood maltreatment and alcohol use problems in emerging adult drinkers. Moreover, the study provides one of the first direct empirical accounts of a conceptual model explaining these relationships, see [24].

The current findings highlight emotion dysregulation and negative urgency as particularly robust correlates of childhood maltreatment and alcohol use problems. Although the findings are cross-sectional, they suggest that individuals who experience difficulties regulating their emotions and who experience heightened urgency, in particular negative urgency, may be at an increased risk for alcohol use problems. Moreover, the findings suggest that experiences of childhood maltreatment may disrupt the development of adaptive emotion regulation processes, contributing to impulsive responses to negative emotions and, in turn, alcohol use problems. While the disruption of emotion regulation as a function of childhood maltreatment could occur through a variety of different channels, parents are often involved [69,70,71]. Of significance, parents play an important role in the development of emotion regulation skills in their children [72,73,74] such that when healthy parental modeling of these skills is lacking, as often occurs in cases of childhood abuse and neglect [75], the child’s ability to regulate their emotions in adaptive ways becomes disrupted, thereby impacting how the child copes with stressors in emerging adulthood [76]. As a result, individuals may act impulsively to offset negative emotional states, such as by using alcohol or other substances, a tendency that is often =more pronounced in emerging adults (e.g., [77,78]).

While we found in the current study a direct effect of childhood maltreatment on reward responsiveness, a finding that is in fact consistent with past work demonstrating blunted responses to rewarding stimuli as a consequence of early life adversity [18,79], our model did not show a relationship between reward responsiveness and impulsivity. This latter negative finding was unexpected based on past research demonstrating a relationship between these factors and a clear relationship between impulsivity and problem substance use. For example, in studies examining the relationship between reward responsiveness and impulsivity on overeating, both factors were found to indirectly predict childhood body mass index through overeating [80]. Likewise, acute stress was found to predict blunted reward processing for participants with heightened behavioral but not trait impulsivity [81]. An explanation for differences between our work and past studies in identifying a relationship between reward responsiveness and impulsivity may involve at least one of several factors, including the statistical models employed, the instruments used to measure the constructs, and/or the demographic characteristics of the samples (e.g., [82]). Also, the differences in findings speak to the importance of replication using similar methodologies and the determination of the extent to which different construct measures reveal similar relationships when statistical models and sample characteristics are held constant.

It is noteworthy that while our model did show a direct effect of childhood maltreatment on reward responsiveness as well as an indirect effect of reward responsiveness on the relationship between childhood maltreatment and alcohol use problems (see Table 3), other measures of the reward construct were without effect. This is in contrast to robust findings demonstrating a strong association between childhood maltreatment, reward-related impairments, and substance use problems [58]. In this regard, a multi-method approach, e.g., testing the model with behavioral in addition to self-report measures of reward and impulsivity, may represent a promising avenue for future research (see Limitations and Future Directions below). Indeed, there is ample evidence in the literature that suggests different types of measures may tap different dimensions of the latent constructs [83,84].

Of note, the current study examined potential variations in the conceptual model based on a dimensional approach that considered the CTQ subscales associated with childhood threat and deprivation separately. Although our findings were consistent whether a cumulative or dimensional approach was taken, prior work has demonstrated differential effects of childhood threat and deprivation on a number of measures, including cognitive and emotional outcomes [11,13]. Also, prior work demonstrated an important role for cultural context in understanding the dimensions of childhood maltreatment [85]. Thus, future work should consider nuances within the model for different outcome measures (e.g., cigarette smoking, illicit drug use, etc.).

Finally, the models that emerged from the current work support existing neurobiological evidence of the effects of childhood maltreatment on emotion dysregulation, reward responsiveness, and impulsivity. For instance, childhood maltreatment has been associated with dysfunction in the left basal ganglia, which is implicated in reward anticipation and learning [18,58,86]; likewise, childhood maltreatment is associated with greater volume in the amygdala, a region of the limbic system involved in emotion regulation [87]. Similarly, lower frontomedial cortical and bilateral insular volumes as well as greater volumes in a region that encompasses the ventral striatum, hypothalamus, and anterior thalamus have been predictably associated with greater impulsive tendencies [23,88].

## 5. Limitations and Future Directions

The findings, while important in helping to establish the validity of the conceptual model, should be interpreted with consideration to several important limitations, including the cross-sectional design, exclusive use of self-report measures, and potential self-selection bias reflected in the sample. First, because this is a cross-sectional study, we cannot make causal inferences, and therefore, the findings should be considered preliminary. Moreover, while the conceptual model allows us to identify associations between variables at a single point in time, we cannot determine the directionality or temporality of these relationships. Thus, replication is needed using a longitudinal design to capture the temporal ordering and dynamic nature of reward, impulsivity, and emotion regulation processes.

Second, the study’s reliance on self-report measures raises the potential for biases related to, for example, social desirability and inaccurate self-perception. As discussed, one way this limitation could be addressed in future research would be by administering both behavioral and self-report measures, wherein the former could directly assess impulsive action, choice, and decision making [83,84]. Moreover, reducing the reliance on a single method would improve the reliability of the results.

Finally, self-selection bias may limit the generalizability of the findings, as part of the sample was recruited via a crowd-sourcing platform (Prolific) and may not reflect the broader population of emerging adults concerning factors such as ethnicity, socioeconomic status, and/or education. Although we collected demographic information on each of these variables in the current study (Table 1), we did not have the statistical power to directly test their influence on the model. Thus, a more detailed consideration of these and other demographic variables is an important avenue for future research.

## 6. Clinical Implications for Practice and Conclusions

The limitations notwithstanding, the current work has the potential to inform prevention and intervention approaches that emphasize, in particular, strategies for addressing negative urgency and emotion regulation. For example, personality-targeted intervention programs can be applied to specifically target the underlying motivation to abuse substances [89], such as the use of alcohol as a coping mechanism for negative affect [90]. Finally, while the findings indeed offer insights for clinical practice, it should be emphasized that longitudinal research is needed to draw more definitive conclusions about their broader applicability in treatment settings.

In sum, this study provides preliminary support for a conceptual model describing associations between factors influencing the relationship between childhood maltreatment and problem substance use, and it informs current research aimed at elucidating the complex mechanisms underlying this relationship in the context of alcohol use problems. Importantly, as this is the first study to empirically test the model, important directions for future research include longitudinal replication and extension to more targeted samples, such as individuals who demonstrate greater impulsive tendencies and/or are diagnosed with substance use disorders. Indeed, by detecting any differences that may exist in how the model behaves based on participant characteristics, this work has the potential to inform clinical intervention and prevention practices (see, for example, [89,91]).

## Figures and Tables

**Table 1:** Demographic data of participants (*n* = 397).

Individual-Level Variables	*n*	*%*
Age *M* = 24.92, *SD* = 2.99		
Biological Sex		
Male	161	40.6
Female	236	59.4
Ethnicity		
European	257	64.7
East or Southeast Asian	42	10.6
South Asian	14	3.5
Middle Eastern	8	2.0
African	22	5.5
Latin	30	7.6
Caribbean	6	1.5
Indigenous	2	0.5
Identified Otherwise	16	4.0
Annual Household Income (USD)		
<10,000	18	4.5
10,000–30,000	50	12.6
30,000–50,000	76	19.1
50,000–70,000	85	21.4
70,000–90,000	65	16.4
90,000–110,000	43	10.8
120,000+	59	14.9
Highest level of education completed by parent(s)		
Neither parent completed high school	164	41.3
Either both parents or one parent completed high school	134	33.8
Either both parents or one parent have some college/university completed	66	16.6
Either both parents or one parent have an undergraduate degree (or higher)	33	8.3

**Table 2:** Descriptive statistics and bivariate correlations (Pearson r) for childhood maltreatment, reward, impulsivity, emotion dysregulation, and substance use problems.

Measure [Range]	Mean	SD	1	2	3	4	5	6	7
1. CTQ [5–125]	43.47	16.38	1						
2. BAS Reward Responsiveness [5–20]	17.10	2.088	−0.207 **	1					
3. BAS Total Score [24–80]	39.48	5.553	−0.147 *	0.742 **	1				
4. SUPPS-Positive Urgency [4–16]	7.66	2.829	0.253 **	−0.078	0.252 **	1			
5. SUPPS-P Negative Urgency [4–16]	9.95	2.871	0.309 **	0.000	0.116 *	0.534 **	1		
6. DERS Total Score [36–180]	90.80	25.177	0.435 **	−0.059	−0.086	0.346 **	0.591 **	1	
7. YAACQ [0–24]	8.03	5.928	0.281 **	−0.031	0.101 *	0.351 **	0.367 **	0.306 **	1

*Note.* For clarity, only the variables used in the final path models are displayed here. CTQ = Childhood Trauma Questionnaire; BAS = Behavioral Activation System; SUPPS-P = Impulsive Behavioral Scale; DERS = Difficulties in Emotion Regulation Scale; YAACQ = Young Adult Alcohol Consequences Questionnaire. Ranges for each of the measures are noted using square brackets. * *p* < 0.05; ** *p* < 0.01.

**Table 3:** Direct and indirect effects of reward-related constructs, impulsivity, and emotion dysregulation variables on the relationship between childhood maltreatment (CTQ) and alcohol use problems (YAACQ).

	Direct Effects of CTQ on Outcome Variables				Direct Effects of Outcome Variables on YAACQ				Indirect Effects of Outcome Variables on the CTQ and YAACQ			
Outcome Variables	*b*	*SE*	*t*	*p*	*b*	*SE*	*t*	*p*	*b*	*SE*	*LLCI*	*ULCI*
TEPS												
Reward Anticipation	−0.118	0.023	−4.387	**<0.001**	−0.008	0.042	−0.189	0.850	0.009	0.005	−0.009	0.011
Reward Consumption	−0.053	0.020	−2.609	**0.009**	0.044	0.053	0.836	0.404	−0.002	0.003	−0.009	0.003
SPSRQ												
Sensitivity to Reward	0.014	0.015	0.9517	0.342	0.360	0.066	5.390	**<0.001**	0.005	0.005	−0.005	0.016
Sensitivity to Punishment	0.095	0.018	5.1542	**<0.001**	0.014	0.054	0.254	0.800	0.001	0.005	−0.008	0.012
BAS												
Reward Responsiveness	−0.026	0.008	−3.497	**0.005**	−0.332	0.163	−2.034	**0.043**	0.009	0.005	**<0.001**	**0.021**
Fun Seeking	−0.010	0.009	−1.111	0.267	0.329	0.134	2.464	**0.014**	−0.003	0.003	−0.011	0.002
Drive	−0.014	0.009	−1.490	0.137	0.389	0.140	2.775	**0.006**	−0.005	0.004	−0.015	0.001
BIS Total Score	0.021	0.015	1.410	0.160	0.175	0.073	2.408	**0.017**	0.004	0.003	−0.001	0.012
SUPPS-P												
Positive Urgency	0.044	0.010	4.326	**<0.001**	0.316	0.122	2.603	**0.010**	0.014	0.006	**0.003**	**0.028**
Negative Urgency	0.054	0.009	5.812	**<0.001**	0.421	0.123	3.435	**<0.001**	0.023	0.008	**0.009**	**0.040**
Sensation Seeking	−0.012	0.010	−1.129	0.260	0.130	0.111	1.169	0.243	−0.001	0.002	−0.006	0.002
Lack of Premeditation	0.037	0.008	4.716	**<0.001**	0.298	0.174	1.720	0.090	0.011	0.007	−0.001	0.027
Lack of Perseverance	0.026	0.008	3.398	**0.007**	−0.151	0.156	−0.969	0.333	−0.004	0.004	−0.012	0.004
DERS Total Score	0.670	0.077	8.673	**<0.001**	0.053	0.013	4.263	**<0.001**	0.036	0.009	**0.018**	**0.055**
CTQ Total Score	-	-	-	-	0.102	0.020	5.046	**<0.001**	-	-	-	-

*Note.* CTQ = Childhood Trauma Questionnaire; TEPS = Temporal Experiences Pleasure Scale; SPSRQ = Sensitivity to Punishment and Reward Questionnaire; BAS = Behavioral Activation System; BIS = Behavioral Inhibition System; SUPPS-P = Impulsive Behavioral Scale; DERS = Difficulties in Emotion Regulation Scale; YAACQ = Young Adult Alcohol Consequences Questionnaire. Bolded values indicate significant associations (*p* < 0.05 for the direct effects).

## Data Availability

The raw data supporting the conclusions of this article will be made available by the authors on request.
